# Recommendations related to occupational infection prevention and control training to protect healthcare workers from infectious diseases: a scoping review of infection prevention and control guidelines

**DOI:** 10.1186/s12913-022-07673-4

**Published:** 2022-03-01

**Authors:** Mohammed O. Qureshi, Abrar A. Chughtai, Holly Seale

**Affiliations:** School of Population Health, University of New South Wales, Level 2, Samuels building, Sydney, Australia

**Keywords:** Healthcare workers, Infection control, Infectious disease transmission, Occupational health, Practice guidelines, Training programs

## Abstract

**Background:**

Events such as the COVID-19 pandemic remind us of the heightened risk that healthcare workers (HCWs) have from acquiring infectious diseases at work. Reducing the risk requires a multimodal approach, ensuring that staff have the opportunity to undertake occupational infection prevention and control (OIPC) training. While studies have been done within countries to look at availability and delivery of OIPC training opportunities for HCWs, there has been less focus given to whether their infection prevention and control (IPC) guidelines adhere to recommended best practices.

**Objectives:**

To examine national IPC guidelines for the inclusion of key recommendations on OIPC training for HCWs to protect them from infectious diseases at work and to report on areas of inconsistencies and gaps.

**Methods:**

We applied a scoping review method and reviewed guidelines published in the last twenty years (2000–2020) including the IPC guidelines of World Health Organization and the United States Centers for Disease Control and Prevention. These two guidelines were used as a baseline to compare the inclusion of key elements related to OIPC training with IPC guidelines of four high-income countries /regions i.e., Gulf Cooperation Council, Australia, Canada, United Kingdom and four low-, and middle-income countries (LMIC) i.e. India, Indonesia, Pakistan and, Philippines.

**Results:**

Except for the Filipino IPC guideline, all the other guidelines were developed in the last five years. Only two guidelines discussed the need for delivery of OIPC training at undergraduate and/or post graduate level and at workplace induction. Only two acknowledged that training should be based on adult learning principles. None of the LMIC guidelines included recommendations about evaluating training programs. Lastly the mode of delivery and curriculum differed across the guidelines.

**Conclusions:**

Developing a culture of learning in healthcare organizations by incorporating and evaluating OIPC training at different stages of HCWs career path, along with incorporating adult learning principles into national IPC guidelines may help standardize guidance for the development of OIPC training programs. Sustainability of this discourse could be achieved by first updating the national IPC guidelines. Further work is needed to ensure that all relevant healthcare organisations are delivering a package of OIPC training that includes the identified best practice elements.

## Background

The importance of regularly attending training programs by healthcare workers (HCWs) has received significant attention in recent years especially during the outbreaks of emerging and re-emerging infectious diseases like Severe acute respiratory syndrome (SARS), Middle East respiratory syndrome coronavirus (MERS-CoV), Ebola and COVID-19 [1–3]. HCWs are at frontline during epidemics and pandemics, and they need to be protected from infectious diseases. However, outside of these emergencies, the level of attention given to training HCWs on occupational infection prevention and control (OIPC) policies and procedures, to reduce their risk of occupational acquired infectious diseases, may not be adequate.

Training HCWs on standard precautions, principles, and practice needs to be implemented to provide the necessary knowledge on compliance to standard precaution practices in order to protect HCWs at work from infectious diseases [4]. A study by John et al. explored this issue with HCWs (doctors, nurses and allied health workers) from eleven north eastern Ohio hospitals and long term care facilities. They examined the frequency, and type of training the HCWs had received, as well as their knowledge and skills on donning and doffing of personal protective equipment (PPE). According to the authors, suboptimal training had been previously delivered around the procedures for PPE, as none of the training methods required the HCWs to demonstrate their level of knowledge or proficiency in the correct use of PPE. 18% (41/222) of participants incorrectly agreed with the statement that there is no need for hand hygiene if gloves are used, suggesting that many HCWs were unaware of the risk for contamination during PPE removal [5]. Other studies on the delivery of OIPC training for HCWs in low and middle-income countries (LMIC) reveal similar findings [6, 7].

OIPC training programs are developed at local level by hospitals or at national level by ministry of health and/or professional organizations like The Society for Healthcare Epidemiology of America [8] or Australasian College for Infection Prevention and Control [9] in Australia. These training programs are often framed based on a set of defined policies and procedures recommended in the national infection prevention and control (IPC) guidelines or by international health organizations like World Health Organization (WHO) and United States, Centers for Disease Control (CDC) [7]. While studies have been done within countries to look at availability and/or delivery of training opportunities, there has been less focus given to the framing and language used to describe staff training within national IPC guidelines. Currently there is little understanding about whether countries classify OIPC training as a core component of national IPC guidelines [10], whether IPC guidelines promote adult learning principles and apply this to the development and delivery of OIPC training [11], whether there is emphasis placed on the review and assessment of staff members within OPIC training programs [10], and lastly about the curriculum covered in OIPC training [10]. Thus in order to identify and map the available evidence a scoping review is conducted in this study [12]. This study thus aimed to examine IPC guidelines of selected health organisations including WHO and CDC with those available within select high income countries and LMIC for the inclusion of recommendations relating to OIPC training programs to protect HCWs from infectious diseases and to report on areas of inconsistencies and gaps.

## Methods

For this study we followed the five stage scoping review process outlined by Levac et al.’s [13]. This scoping review did not involve primary research with human subjects and therefore did not warrant institutional ethical approval. This scoping review is registered with the Open Science Framework (https://osf.io/ryk9b).

### Stage 1: identifying the research questions

The following questions guided this scoping review for the inclusion of OIPC training related information in the IPC guidelines: What is the recommendation on framework of OIPC training programs? What is the recommendation on mandatory vs voluntary participation of HCWs in OIPC training programs? What is the recommendation on evaluation of OIPC training programs? What is the recommendation on frequency and delivery of OIPC training program? What is the recommendation on topics covered for OIPC training programs?

### Stage 2: identifying relevant studies

Between January 2020 to September 2020, two strategies were used to identify IPC guidelines of international agencies, countries/region selected for this study. Firstly, the websites of international public health agencies such as WHO, CDC and selected country health departments/Ministry of Health/Department of public health including four high-income countries/regions i.e. Australia [14], Canada [11], United Kingdom (UK) [15] and Gulf Cooperation Council (GCC) and [16] four LMIC i.e. India [17], Indonesia [18], Pakistan [19] and Philippines [20] were searched so that comparisons could be made with the IPC guidelines of WHO and CDC for the inclusion of key elements related to OIPC training. We selected two international guidelines of WHO and CDC as they are globally used as a reference for policy and guideline development [10, 21]. The main reason for including the guidelines of UK, Australia and Canada was that these countries were included in a list of 11 high performing healthcare systems [22]. GCC IPC guideline was chosen as it represented high-income countries where there had been the emergence of an infectious disease i.e. MERS-CoV [23]. The four LMIC was included as they account for 25% of the world’s population and represent countries where expatriate HCWs originate from and are recruited by all high-income countries [24–27].

Then, a key word search was conducted using Google, with the first 10 results per page and the first two pages of hits reviewed. We did not impose any language restriction. The publicly available national IPC guidelines of all the selected countries were in English language except Indonesia. Google translate was used to translate the Indonesian IPC guideline in English language and reviewed inhouse for accuracy by a native Indonesian colleague. Key words used for search were semantically related and grouped into four categories: (1) ‘infection control’, infection prevention and control’, ‘occupational infection prevention and control’, ‘occupational health and safety’, ‘workplace health and safety’, ‘occupational health services’, ‘occupational health’, ‘infectious diseases transmission’. 2) ‘guidelines’, ‘policies’, ‘program’, ‘code of practice’, ‘manual’, ‘regulations’, ‘practice guidelines’, ‘training guidelines’; (3) ‘healthcare’, ‘national health care facility’, ‘acute health care facility’ and (4) ‘healthcare personnel’, ‘healthcare workers’, ‘healthcare providers’. Boolean operators were used to combine keywords i.e., AND/OR/*. Results from the search then were refined using the ‘AND’ Boolean operator with the selected 10 organization/country names.

### Stage 3: identifying the study selection criteria

**Fig. 1:**
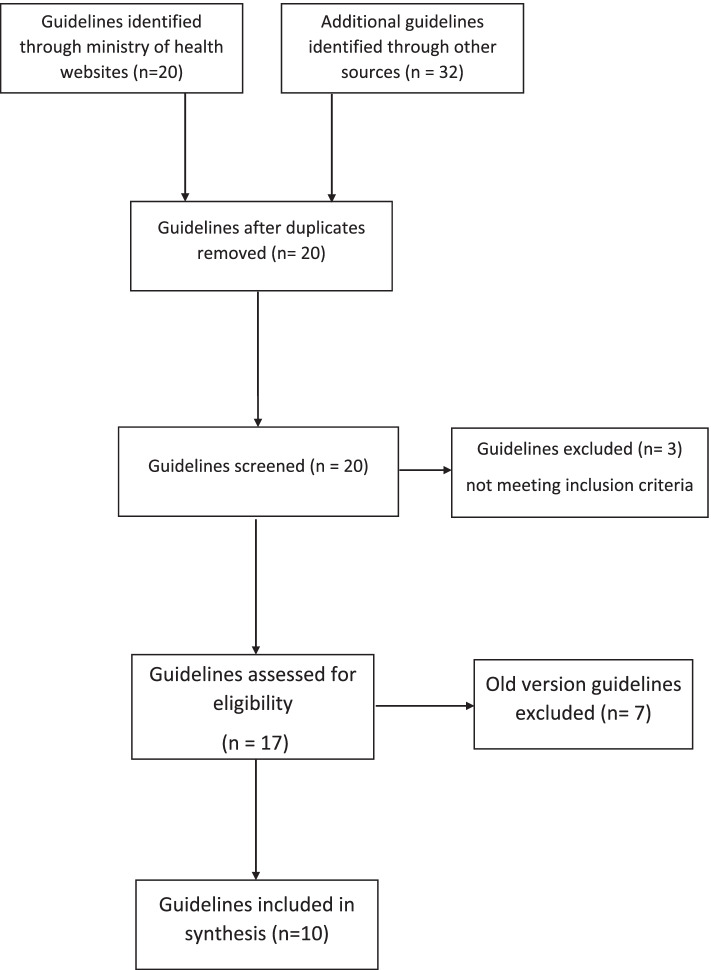
Study flow chart

IPC guidelines of the selected international agencies and countries published in the last twenty years (2000–2020) were screened for GRADE (Grading of Recommendations, Assessment, Development and Evaluations) and latest version. GRADE is the most widely adopted tool for grading the quality of evidence and for making recommendations with over 100 organisations worldwide officially endorsing it [28]. If two versions of the same document were found, the most recent version was included. National IPC guidelines were selected over individual facilities when more than one guideline was found in a country (see Fig. 1).

### Stage 4: charting the data

WHO and CDC guidelines were used as a baseline and reviewed to extract the key elements of OIPC training including, application of adult learning principles; evaluation of training programs (review of training programs and assessment of HCWs); mandatory attendance; frequency and delivery of training at undergraduate/post graduate education and/or on joining a healthcare setting (orientation and/or induction), job specific training, outbreak control training; methods of delivery of training like, on the job training, e-learning, simulation training, bed side training, retraining,problem based training, hands-on workshops, focus groups, peer-to-peer training, class room based training, and topics covered in OPIC training program. These key elements were used to form an audit tool to help with extracting information from the other selected IPC guidelines. Key elements were first assessed by the lead author (MOQ) and then validated by co-authors (HS and AAC). Extracted information was then coded and assigned appropriate themes [29]. The lead author (MOQ) assigned codes to themes which were then validated by the co-authors (HS and AAC). Discrepancies were discussed and resolved. The following baseline data related to title and summaries were also extracted from each IPC guidelines: issuing organization/country, department, document title and year of publication/ revision date (see Table 1).

**Table 1:** Base line data of all Infection prevention and control guidelines along with regional distribution and economic status of countries as per World Health Organization

Serial Number	Issuing Organization or Country	WHO Region	Economic status	Organization	Document Title	Published/Revised Year
1	**World Health Organization**	Not applicable	Not applicable	WHO	Guidelines on Core Components of Infection Prevention and Control Programmes at the National and Acute Health Care Facility Level. [10]	2016
2	**United States of America, Centre for Disease Control**	Americas	High income country	Centers for Disease Control and Prevention National Center for Emerging and Zoonotic Infectious Diseases Division of Healthcare Quality Promotion. United States of America	Infection Control in Healthcare Personnel: Infrastructure and Routine Practices for Occupational Infection Prevention and Control Services [30]	2019
3	**Australia**	Western Pacific	High income country	National Health and Medical Research Council	Control of Infection in Healthcare Australian Guidelines for the Prevention and Control of Infection in Healthcare Australian Guidelines for the Prevention and Control of Infection in Healthcare [14]	2019
4	**Canada**	Americas	High income country	Infection Prevention and Control Canada (IPAC Canada)	Infection Prevention and Control (IPAC) Program Standard [11]	2016
5	**Gulf Cooperation Council**	East Mediterranean	High income economy	GCC Center for infection control, Ministry of National Guard, Health affairs, Kingdom of Saudi Arabia	The GCC Infection Prevention and Control Manual 3rd Edition [16]	2018
6	**United Kingdom**	European	High income country	Department of Health	The Health and Social Care Act 2008: Code of Practice on the prevention and control of infections and related guidance [15]	2015
7	**India**	South-East Asia Region	Medium–Low income country	National Centre for Disease Control, Directorate General of Health Services Ministry of Health and Family Welfare, Government of India	National guidelines for infection prevention and control in healthcare facilities [17]	2020
8	**Indonesia**	South-East Asia	Medium income country	The Minister of Health, Republic of Indonesia	Peraturan menteri kesehatan republik Indonesia [18]	2017
9	**Pakistan**	East Mediterranean	Medium–Low income country	National Institute of Health	National Guidelines Infection Prevention & Control [19]	2020
10	**Philippines**	Western Pacific	Medium–Low income country	National Center for Health Facility Development, Department of Health	National Standards for Infection Control for Healthcare Facilities [20]	2009

*WHO* World Health Organisation

### Stage 5: summarizing results

**Fig. 2:**
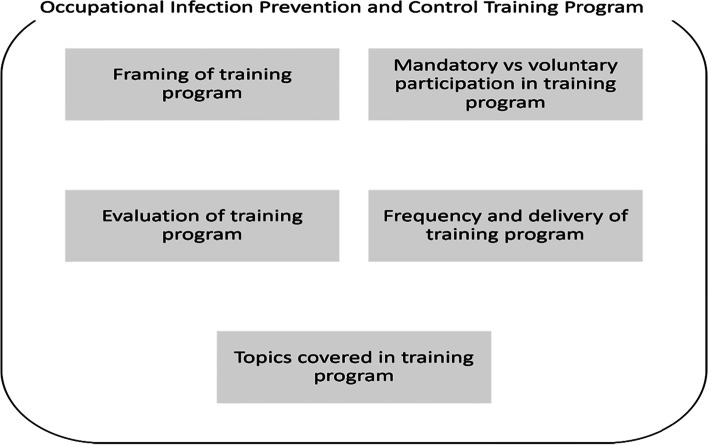
Graphic representation of the key occupational infection prevention and control training program themes identified from the infection prevention and control guidelines

The results were organised under the following five themes: framing of OIPC training program, mandatory vs voluntary participation in OIPC training program, evaluation of OIPC training program, frequency and delivery of OIPC training program, and topics covered in OIPC training program (see Fig. 2).

## Results

We reviewed 10 IPC guidelines for analysing information related to OIPC (IPC) training. We reviewed IPC guidelines of two globally recognised public health agencies including WHO [10] and CDC [30]. We also reviewed IPC guideline of one regional agency namely, GCC [16], three high-income countries including, Australia [14], Canada [11] and United Kingdom [15]) and four LMIC including India [17], Indonesia [18], Pakistan [19] and Philippines [20]. Except for the Filipino IPC guideline, all the other guidelines were developed in the last five years and followed a formal grading system e.g. GRADE or a system that incorporated elements of the GRADE approach, to show the strength of the recommendations underpinning the guideline. Table 1 provides details of the included guidelines.

### Framing of OIPC training program

The WHO as a part of its multimodal strategy, recommends embedding a culture of infection prevention within the establishment for safe working conditions of the facility. This language is also reflected in the IPC guidelines of CDC, Australia, Canada, India, and Pakistan. The Canadian guidelines goes one step further to also outlines the mechanism for providing a safe working environment by recommending conducting ongoing training programs for HCWs and others working in the healthcare settings. The WHO and CDC, IPC guidelines, recommended that healthcare facilities focus on core components of IPC, of which OIPC training of HCWs should be one. At the country level, all the guidelines except that of UK, outlined HCWs training and education as a core component of IPC and only Pakistan’s IPC guideline included a chapter or a section on ‘education and training’ in their IPC manual.

All the IPC guidelines use the terms education and training interchangeably. All IPC guidelines suggest that the aim of OIPC training is to equip HCWs with requisite infection control skills needed to perform their job efficiently and smoothly.

### Mandatory vs voluntary participation in OIPC training program

Both the WHO and CDC IPC guidelines recommend that OIPC training should be provided to HCWs before being allowed to perform their duties and after they join a healthcare setting. The WHO guideline, however, does not directly refer to the session as ‘mandatory’, nor does the UK national IPC guidelines. Whereas, just like CDC, the IPC guidelines of Canada, GCC, Pakistan, India, Indonesia, and Australia emphasize mandatory attendance of HCWs. In Canada only some provinces mandate periodic OIPC training programs for all HCWs.

### Evaluation of OIPC training program

The WHO recommends periodic evaluations of the effectiveness of OIPC training programs and the assessment of staff knowledge be undertaken on a routine basis. The GCC guideline suggests establishing an OIPC hospital-based certification policy to ensure that HCWs knowledge and skills are reviewed and renewed regularly. Whereas the CDC guideline does not include any reference to the evaluation of OIPC training programs. Similarly, the guidelines of UK, Indonesia, and Pakistan, also do not provide any recommendations regarding the evaluation of OIPC training programs. Indian and Indonesian guidelines only focus on the assessment of HCWs for IPC practices. Indonesia’s IPC guidelines recommend monthly auditing of training programs. It is not however clear if auditing also involves reviewing training programs, assessment of HCWs knowledge or both. All the guidelines recommend reviewing their IPC manual on a regular basis.

### Frequency and delivery of OIPC training program

WHO and Australian guidelines recommend OIPC training as part of undergraduate and post graduate education. WHO recommends that OIPC training be conducted at orientation and then yearly for HCWs, which also aligns with the recommendations from CDC, Australia, Canada and Pakistan. The GCC guidelines on the other hand recommend that it occurs every two years while the Philippine’s guideline suggests conducting it at least twice a year. Indian and Indonesian guidelines both recommend OIPC training at orientation but provide no further comment beyond that. UK and Pakistan’s guidelines also suggested OIPC training at induction. All the guidelines outlined job specific OIPC training. WHO and CDC guidelines recommends outbreak control OIPC training for HCWs, which is also reflected in the guidelines of Canada, UK and India.

The WHO guidelines outlines 10 different methods for the delivering of training to HCWs namely, on the job or in-service training, oral instructions, e-learning, simulation, bed side training, problem based learning, hands on workshops, focus groups, peer-to-peer training, classroom based simulation, whilst the CDC guideline specifies only two, namely, on the job or in-service training and retraining. Australian guideline only refers to three namely, on the job or in-service training, e-learning, and hand-on workshops as methods of delivery of training HCWs. Except Simulation, retraining and classroom based training, Canadian guidelines recommends all the other methods of training programs suggested in the WHO guideline. GCC guidelines outlines on the job or in-service training and simulation only. Indonesian and Philippines guidelines recommend e-learning, while as, Pakistan’s guideline recommends hands-on workshop as method of delivery of training HCWs. For OIPC training based on HCWs work status and method of delivery, information was sought but nothing related was found in most of the guidelines.

### Topics covered in OIPC training program

The topics suggested for inclusion in OIPC training vary across guidelines. Like the WHO and CDC, the guidelines of Australia, Canada and GCC recommend tailoring the training curriculum to national and local requirements but emphasise that hospitals include the basic minimum curriculum suggested in their respective guidelines as a part of the training module. Canadian and GCC guidelines suggests that training programs should be based on adult teaching–learning principles, whereas no other guideline mentions about this including WHO and CDC. The Australian guidelines in addition to recommending a minimum number of topics for OIPC training for on-the-job HCWs also suggests including up-to-date information on IPC basics, policy, procedures, quality assurance and incident monitoring, for health-related courses at undergraduate and postgraduate academic level. Further information is included in Table 2.

**Table 2:** Curriculum recommended for occupational infection prevention and control training programs

Organization / Country	Training Curriculum
World Health Organization	Refer to international curricula and networks for specialized infection prevention and control programmes and to adapt these documents and approaches to national needs and local available resources
United States of America, Centre for Disease Control	• Federal, state, and local education and training requirements • Modes of infectious disease transmission and implementation of standard and transmission-based precautions • Hand hygiene • Sharps injury prevention • Immunizations recommended by the Advisory Committee on Immunization Practices (ACIP) for healthcare personnel • Healthcare personnel screening for selected infectious diseases before job placement and periodically thereafter • How to access occupational health services, when needed, and expectations for reporting exposures • Expectations for reporting illnesses or conditions (work-related or acquired outside of work), such as rashes or skin conditions (e.g., non-intact skin on hands); febrile, respiratory, and gastrointestinal illnesses, and hospitalizations resulting from infectious diseases • Sick leave and other policies and procedures related to infectious healthcare personnel, including the risks of presenteeism to other healthcare personnel and patients
Australia	• An understanding of the modes of transmission of infectious agents and of risk management • Effective work practices that minimise the risk of transmission of infectious agents • Governance structures that support the implementation, monitoring and reporting of infection prevention and control work practices • Compliance with legislation, regulations and standards relevant to infection control
Canada	• Critical IPC assessment skills / risk assessment • IPC program basic standards of practice (“core competencies”): • hand-hygiene for staff, service providers, and volunteers • concepts of Routine Practices • concepts of Additional Precautions • appropriate use of PPE •safe management of sharps • health care worker immunization • work restrictions due to infectious diseases • equipment cleaning and disinfection/sterilization • environmental cleaning • basic microbiology and transmission of microorganisms • how and when to report IPC-related incidents, injuries and issues of concern • information on common HAIs affecting the organization (e.g., methicillin-resistant Staphylococcus aureus, vancomycin-resistant enterococci, Clostridium difficile infection, device-associated infections); and • Additional IPC resources available, both within and outside the organization
Gulf Cooperation Council	• Hand hygiene • Donning and doffing of personal protective equipment
United Kingdom	No specific Information
India	• Information on modes of transmission of infectious diseases, level of occupational risk (to reduce fear of contact with infected patients) prevention and control • Safe work practices • Handling of PPE and clothing • Reporting of exposure incident • Techniques on stress management, provision of appropriate staffing levels, shift, rotation, counselling, support and communication skills • Regulations and policies
Indonesia	• Basic principles of IPC • Hand hygiene • Cough ethics • Waste handling • Appropriate use of PPE
Pakistan	• Infection prevention control • Personal hygiene • Management of sharps injuries and exposure to blood and body fluids
Philippines	• Epidemiology of healthcare associated infections • Hand hygiene • Isolation precautions • Decontamination • Disinfection & Sterilization • Care of the environment and hospital waste management • Infection control during routine patient care • Infection control in special and high-risk area • Infection control in hospital ancillary services • Healthcare worker infection risks and prevention

## Discussion

From this review we found similarities, variations, and omissions in the way OIPC training programs for HCWs are framed across high-income countries and LMIC. While all the guidelines suggest OIPC training be delivered to HCWs, there is a difference in how frequently it should be delivered. For example, the WHO [10], Canada [11], Pakistan [19], and Australian [14] guidelines recommend conducting it on yearly basis, whereas the GCC guidelines [16] recommends it every two years, the Philippines guideline [20] suggests conducting it biannually, whilst the Indian [17] and Indonesian [18] guidelines do not comment on how frequently it should be conducted. None of the country IPC guidelines include the same level of detail as the WHO. In addition, none of the policies and guidelines from the selected focus countries promoted the involvement of HCWs in the development of OIPC training programs even though it would help develop staff centric training programs.

There are significant differences in the OIPC topics, covered in the guidelines of high and LMIC countries. The WHO [10] recommends that individual countries decide the curriculum of the IPC training programs, something which is reflected in the CDC [30] and Australian IPC guidelines [14]. The authors of this study believe that the observed heterogeneity in IPC training curriculum and frequency at which it should be delivered can be eliminated through the development of common learning objectives and development of core competencies when it comes to OIPC training. The baseline set of topics should include information on modes of transmission of infectious diseases, occupational practices that minimize the risk of transmission of infectious diseases like, practicing correct hand hygiene & respiratory hygiene, appropriate use of PPEs, medication storage and handling, proper equipment cleaning and disinfection, sharps injury prevention, immunizations for HCWs, techniques of stress management for HCWs and HCWs screening for infectious diseases at starting a new job and periodically thereafter. Information about compliance with legislation and regulations relevant to infection control should also be included like sick leave and other policies and procedures related to infectious HCW including the risks of continuing working in spite of being infectious to other HCWs and patients. This should also include information about reporting exposure incidents, injuries and issues of concern by HCWs to relevant healthcare authorities.

While education and training are absolutely related, they are by no means the same process. Education refers to the gaining of comprehensive knowledge and skills usually at the college or university level, while training refers to the acquisition of job specific and applied knowledge and skills [31]. In all the IPC guidelines, the terms ‘education’ and ‘training’ have been used interchangeably. We found that OIPC education at undergraduate or post graduate level were recommended only in Australian IPC guidelines [14] and none of the other guidelines included any reference to it. OIPC training at undergraduate or post-graduate education are intended to provide students in the health domain with a basic solid education on IPC principles and help them protect themselves from the threat of infectious diseases as HCWs.

The impact of education and training programs depends a great deal on the background experience and prior learning of the employee and what they bring to any new learning process. It is thus critical that learning approaches recognise the background and diversity in the content and style of approach, which requires the integration of adult learning principles in the development and delivery of training programs [32, 33]. Malcolm Knowles the pioneer of adult learning identified six principles of adult learning for helping adults learn better which include: (1) giving adults the freedom to assume responsibility for their own choices; (2) encouraging learners to connect past experiences with current knowledge and activities; (3) aligning the learning activities so that the goals of learners are fulfilled; (4) relating the assigned tasks to adult learners own learning goals; (5) applying the theoretical concepts learned inside the classroom into real-life situations and (6) acknowledging learners contribution [34]. From our study, we found that only two guidelines from high income countries and region namely the Canadian [11] and GCC guidelines [16] suggested the application of adult learning principles in the development and delivery of OIPC training. To ensure a learner centric approach, it is important that IPC guidelines should consider recommending the use of adult learning principles for developing OIPC training programs for HCWs.

In order to ensure consistency with current guidelines and best practices, performance standards can be achieved if OIPC training programs delivered to HCWs are periodically evaluated [35]. When it comes to evaluation of OIPC training programs, there are noticeable differences between the IPC guidelines of the selected focus countries. While IPC guidelines of the WHO [10] and all the high-income countries (except UK) emphasise the need for evaluation of OIPC training programs, the CDC guidelines [30], and the guidelines of two LMIC (Pakistan [19] and Indonesia [18]) do not discuss this. Furthermore, low income-countries like India [17] and Philippines [20] recommend assessing the IPC knowledge of HCWs to demonstrate competency, the GCC guidelines necessitates HCWs to qualify the OIPC training program with a minimum of 80% passing score. It is recommended that periodic evaluation of OIPC training programs should be included in the guidelines, including encouraging organisations to seek feedback from HCWs about the quality of the training, as well as assessing HCWs on their understanding and skills. Again, guidelines need to be explicit on how frequently HCWs should be assessed and the approaches that can be used, drawing on what is known regarding best principles from the literature.

A major project launched in 2013 by the European Centre for Disease Prevention and Control (ECDC) and called by the name of ‘Implementation of a training strategy for infection control in the European Union’ emphasises the importance of attending OIPC training by HCWs [36]. However, in the current study, WHO [10] and UKs [15] IPC documents do not elaborate on the mandatory attendance of OIPC by HCWs. In comparison to high income countries, all the LMIC recommend mandatory attendance of OIPC training programs by HCWs. GCC guidelines [16] asserts on establishing a mandatory hospital-based IPC certification policy to ensure that HCWs knowledge and skills are updated regularly. Philippines IPC guidelines [20] recommends on establishing a mechanism for monitoring compliance. Indian [17] and Australian IPC guidelines [14] suggest a possible disciplinary action against HCWs as a mechanism for necessitating adherence to policies and guidelines.

### Limitations

A limitation of this study is that our findings are not representative of all high-income countries and LMIC. We also acknowledge that there could be other topic specific IPC guidelines adhered by OIPC professionals. Also, countries have different capabilities, so the depth of IPC guidelines would differ from recommendations outlined in WHO and CDC guidelines. Additionally, scoping review of IPC guidelines updated before and after the COVID-19 pandemic could have been conducted.

## Conclusion

The policies and procedures defined in the international and local IPC guidelines steer the development of OIPC training programs for HCWs. In effect, successful delivery of OIPC training in healthcare settings depend on the recommendations present in national and international IPC guidelines such as WHO and CDC. From the review, significant inconsistencies were identified between the recommendations suggested by the selected guidelines of international health organizations, high- and LMIC. Although all the guidelines suggest OIPC training be provided to HCWs, however, not all the IPC guidelines include recommendations on the key elements of OIPC training outlined by WHO and CDC IPC guidelines, which may lead to undesirable outcomes like poor delivery of quality OIPC training programs. Training alone will not change the behaviour of HCWs and is only one of the ways to protect HCWs from acquiring infectious diseases, however, findings from this study highlight the importance of developing a culture of learning in the healthcare organizations by integrating adult learning principles into the development of OIPC training modules. There is also a need for outcome focused training evaluation framework to capture whether the OIPC training programs have had an impact on HCWs attitudes, understanding and practice. If not, then revisions need to be made to the approach and/or content of the training programs. A competent OIPC training program should entail key elements listed in the IPC guidelines like training curriculum, frequency of training program, mandatory attendance, delivery method, monitoring, and evaluation of training program. Sustainability of this discourse could be achieved by updating the international, national and regional IPC guidelines. Further studies should be followed to explore guidelines available in countries that share a similar socio-economic status.

## Data Availability

All data generated or analysed during this study are included in this published article.

## Abbreviations

CDC: United States Centers for Disease Control
ECDC: European Centre for Disease Prevention and Control
GCC: Gulf Cooperation Council
GRADE: Grading of Recommendations, Assessment, Development and Evaluations
HCWs: Healthcare Workers
IPC: Infection Prevention and Control
LMIC: Low and Middle-Income Countries
MERS-CoV: Middle East Respiratory Syndrome Coronavirus
OIPC: Occupational Infection Prevention and Control
PPE: Personal Protective Equipment
SARS: Severe Acute Respiratory Syndrome
UK: United Kingdom
WHO: World Health Organization

## References

[1] Hageman JC (2016). Infection prevention and control for Ebola in health care settings—West Africa and United States. MMWR Suppl.

[2] Suwantarat N, Apisarnthanarak A (2015). Risks to healthcare workers with emerging diseases: lessons from MERS-CoV, Ebola, SARS, and avian flu. Curr Opin Infect Dis.

[3] Huh S (2020). How to train health personnel to protect themselves from SARS-CoV-2 (novel coronavirus) infection when caring for a patient or suspected case. J Educ Eval Health Prof.

[4] Asmr Y, Beza L, Engida H, Bekelcho T, Tsegaye N, Aschale Y (2019). Assessment of Knowledge and Practices of Standard Precaution against Blood Borne Pathogens among Doctors and Nurses at Adult Emergency Room in Addis Ababa. Ethiopia Emergency Medicine International.

[5] John A, Tomas ME, Cadnum JL, Mana TSC, Jencson A, Shaikh A (2016). Are health care personnel trained in correct use of personal protective equipment?. Am J Infect Control.

[6] Phukan P (2014). Compliance to occupational safety measures among the paramedical workers in a tertiary hospital in Karnataka, South India. Int J Occup Environ Med.

[7] John A, Tomas ME, Hari A, Wilson BM, Donskey CJ (2017). Do medical students receive training in correct use of personal protective equipment?. Med Educ Online..

[8] SHEA. Society for healthcare epidemiology of America publishes infection control education course 2020 [cited 2021 02 June]. Available from: https://www.shea-online.org/index.php/journal-news/press-room/press-release-archives/753-society-for-healthcare-epidemiology-of-america-publishes-infection-control-education-course.

[9] ACIPC. Foundations of IPC 2021 [cited 2021 02 June]. Available from: https://www.acipc.org.au/education/foundations/.

[10] World Health Organization W (2016). Guidelines on core components of infection prevention and control programmes at the national and acute health care facility level WHO.

[11] Canada. IPaCI (2016). Infection prevetion and control (IPAC) program standard. Can J Infect Control.

[12] Munn Z, Peters MD, Stern C, Tufanaru C, McArthur A, Aromataris E (2018). Systematic review or scoping review? Guidance for authors when choosing between a systematic or scoping review approach. BMC Med Res Methodol.

[13] Levac D, Colquhoun H, O'Brien KK (2010). Scoping studies: advancing the methodology. Implement Sci.

[14] NHMRC. Australian guidelines for the prevention and control of infection in healthcare Australian guidelines for the prevention and control of infection in health: National Health and Medical Research Council; 2019 [cited 2020 20 June]. Available from: https://www.nhmrc.gov.au/about-us/publications/australian-guidelines-prevention-and-control-infection-healthcare-2019.

[15] Health Do. The health and social care act 2008. Code of practice on the prevention and control of infections and related guidance 2015 [cited 2020 20 May]. Available from: https://assets.publishing.service.gov.uk/government/uploads/system/uploads/attachment_data/file/449049/Code_of_practice_280715_acc.pdf.

[16] CIC GCC. The GCC infection prevention and control manual Gulf Cooperation Council – Center for infection control; 2018 [cited 2020 20 June]. 3rd Edition:[Available from: http://gdipc.org/wp-content/uploads/2018/07/The-GCC-Infection-Prevention-and-Control-Manual-3rd-Edition.pdf.

[17] NCDC. National guidelines for infection prevention and control in healthcare facilites: National Centre for Disease Control, Directorate General of Health Services Government of India; 2020 [cited 2020 20 May]. Available from: https://www.mohfw.gov.in/pdf/National%20Guidelines%20for%20IPC%20in%20HCF%20-%20final%281%29.pdf.

[18] Kesehatan M. Pedoman pencegahan dan pengendalian infeksi di fasilitas pelayanan Kesehatan 27 ed: Menteri Kesehatan Republik Indonesia; 2017.

[19] NIH. National Guidelines Infection Prevention & Control National Institute of Health, Pakistan; 2020 [cited 2020 30 May]. Available from: https://www.nih.org.pk/wp-content/uploads/2020/04/Complete_IPC_Guideliens.pdf.

[20] NCHFD NCfHFD-. National Standards in Infection Control for Healthcare Facilities (Revised Edition): Department of Health, Phillipines Hospital Infection Control Society (PHICS); 2009 [cited 2020 20 May]. Available from: https://www.doh.gov.ph/node/10061.

[21] Chughtai AA, Seale H, MacIntyre CR (2013). Availability, consistency and evidence-base of policies and guidelines on the use of mask and respirator to protect hospital health care workers: a global analysis. BMC Res Notes.

[22] Schneider EC, Sarnak DO, Squires D, Shah A (2017). Mirror, Mirror 2017: nternationa Comparison Ref ects F aws and Opportunities for Better US Hea th Care.

[23] World Health Organization (WHO). MERS Situation update November 2018 2018 [cited 2019 Jan 07]. Available from: http://applications.emro.who.int/docs/EMROPub_2018_EN_20768.pdf?ua=1&ua=1.

[24] Thompson M, Walton-Roberts M (2019). International nurse migration from India and the Philippines: the challenge of meeting the sustainable development goals in training, orderly migration and healthcare worker retention. J Ethn Migr Stud.

[25] Boldbaatar A (2020). International nurse migration: impact on low-and middle-income source countries and policy responses.

[26] Hossain N, Shah N, Shah T, Lateef SB (2016). Physicians' Migration: perceptions of pakistani medical students. J Coll Physicians Surg Pak.

[27] Tangcharoensathien V, Travis P, Tancarino AS, Sawaengdee K, Chhoedon Y, Hassan S (2018). Managing In- and Out-Migration of Health Workforce in Selected Countries in South East Asia Region. Int J Health Policy Manag.

[28] Guyatt GH, Oxman AD, Vist GE, Kunz R, Falck-Ytter Y, Alonso-Coello P (2008). GRADE: an emerging consensus on rating quality of evidence and strength of recommendations. BMJ.

[29] Braun V, Clarke V (2012). Thematic analysis.

[30] Kuhar DT, Carrico RM, Cox K, de Perio MA, Irwin KL, Lundstrom T, et al. Infection control in healthcare personnel: infrastructure and routine practices for occupational infection prevention and control services. 2019. https://stacks.cdc.gov/view/cdc/82043.

[31] Dearden R (1984). Education and training. Westminst Stud Educ.

[32] Collins J (2004). Education techniques for lifelong learning: principles of adult learning. Radiographics.

[33] Bryan RL, Kreuter MW, Brownson RC (2009). Integrating adult learning principles into training for public health practice. Health Promot Pract.

[34] Hartree A (1984). Malcolm Knowles’ theory of andragogy: A critique. Int J Lifelong Educ.

[35] Ahmad I, ud Din  S (2009). Evaluating training and development. Gomal J Med Sci.

[36] Brusaferro S, Arnoldo L, Cattani G, Fabbro E, Cookson B, Gallagher R (2015). Harmonizing and supporting infection control training in Europe. J Hosp Infect.

[37] NHMRC. National Statement on Ethical Conduct in Human Research2007 (Updated 2018): National Health and Medical Research Council, the Australian Research Council and Universities Australia.; 2018 [cited 2021 18 Aug ]. Available from: https://www.nhmrc.gov.au/about-us/publications/national-statement-ethical-conduct-human-research-2007-updated-2018#toc__556.

